# ALLin4IPE- an international research study on interprofessional health professions education: a protocol for an ethnographic multiple-case study of practice architectures in sites of students’ interprofessional clinical placements across four universities

**DOI:** 10.1186/s12909-024-05902-4

**Published:** 2024-08-28

**Authors:** Annika Lindh Falk, Madeleine Abrandt Dahlgren, Johanna Dahlberg, Bente Norbye, Anita Iversen, Kylie J. Mansfield, Eileen McKinlay, Sonya Morgan, Julia Myers, Linda Gulliver

**Affiliations:** 1 Linköping University, Linköping, Sweden; 2 UiT The Arctic University of Norway, Tromsø, Norway; 3 University of Wollongong, Wollongong, Australia; 4 University of Otago, Dunedin, New Zealand

**Keywords:** Clinical placement, Ethnographic field studies, Health professions education, International multiple case study, Interprofessional education, Interprofessional learning, Participatory observations, Practice architectures, Practice theory

## Abstract

**Background:**

The global discourse on future health care emphasises that learning to collaborate across professions is crucial to assure patient safety and meet the changing demands of health care. The research on interprofessional education (IPE) is diverse but with gaps in curricula design and how IPE is enacted in practice.

**Purpose and aims:**

This research project will identify. 1) how IPE in clinical placements emerges, evolves, and is enacted by students when embedded in local health care practices, 2) factors critical for the design of IPE for students at clinical placements across the four countries.

**Methods:**

A study involving four countries (Sweden, Norway, Australia and New Zealand) using the theory of practice architectures will be undertaken between 2023 and 2027. The project is designed as an international, collaborative multiple-case ethnographic study, using the theoretical framework of practice architectures (TPA). It will include four ethnographic case studies of IPE, one in each country. Data will be collected in the following sequence: (1) participant observation of students during interprofessional placements, (2) interviews with students at clinical placement and stakeholders/professionals, (3) Non-clinical documents may be used to support the analysis, and collection of photos may be use as memory aids for documenting context. An analysis of “sayings, doings and relatings” will address features of the cultural- discursive, material-economic, social-political elements making up the three key dimensions of TPA. Each of the four international cases will be analysed separately. A cross case analysis will be undertaken to establish common learning and critical IPE design elements across the four collaborating universities.

**Discussion:**

The use of TPA framework and methodology in the analysis of data will make it possible to identify comparable dimensions across the four research sites, enabling core questions to be addressed critical for the design of IPE. The ethnographic field studies will generate detailed descriptions that take account of country-specific cultural and practice contexts. The study will also generate new knowledge as to how IPE can be collaboratively researched.

## Background

The global discourse on future health care emphasizes interprofessional collaborative capability as being crucial to meet changing demands on health care systems. These demands are the result of aging populations, increasing inequities in health care outcomes, the increasing number of those with complex health conditions and shortage of health care personnel [1, 2]. The World Health Organisation (WHO) [2] states interprofessional education (IPE) *“occurs when students from two or more professions learn about*,* from and with each other to enable effective collaboration and improve health outcomes”* (p. 10), signalling that IPE involves interaction between students in learning activities. When the students understand the value of collaborative practice, they are better prepared to become a member of the collaborative practice team and provide better health services. The rationale for IPE, according to WHO [2] is that health professions should strive to design IPE activities to develop and optimize students’ collaborative competences to prepare them for the above challenges in their future working life [2], something that is also emphasized in the Winterthur/Doha declaration of Interprofessional. Global 2023 [3].

Efforts to explore IPE from the international research community are rapidly growing [4]. Meta-analyses and scoping reviews of IPE initiatives indicate a diverse picture of IPE programmes [5–7]. Vuurberg et al. [8], in their review of research studies on IPE between 1970 and 2017, point to a paucity of research regarding the influence of collaborative work on the development of professional interpersonal skills. In recent years it has been argued that there is a potential to offer IPE in clinical placements thus providing authentic learning opportunities for students in the context of complex health care practices [9]. Interprofessional learning during clinical placements is a step forward to develop and strengthen students’ interprofessional competencies, professional identity, and confidence [10, 11].

Several reviews regarding students’ perceptions about IPE in clinical placements mostly report positive experiences, e.g., increased communication skills and increased knowledge of each other’s roles [12–14]. Results also indicate increased abilities with regard to working within a team and improved communication [e.g., 15–16]. Longer periods of IPE activities seem to strengthen students’ professional identity formation and overcome traditional hierarchical prejudices that can exist in interprofessional teams [e.g., 17–18]. On the other hand, it has also been suggested [19] that the lack of attention to power and conflict in the IPE literature might indicate a neglect of the impact of organizational, structural and institutional issues; and thereby might veil the very problems that IPE attempts to solve.

Published examples of IPE activities in clinical placements have covered a wide range of types of activities as well as numbers of hours and days. Initiatives have been developed that extend over a few hours or a day. Students taking responsibility for a team round in clinical placement [20] or structured interprofessional workshops about falls prevention [21], are both examples of formal activities arranged during clinical placement periods. A workplace-driven, informal, arrangement where students on uni-professional clinical placement were engaged in interprofessional teamwork for one day [22] is another example. Interprofessional activities where students practice together for a longer period have been developed and implemented during the past 25 years. Interprofessional training wards where students work together, often for a period of around two weeks, with the overall responsibility for patients’ care, have been a successful activity developed worldwide [23–25]. The heterogeneity of activities, educational approaches, and outcome measures, makes it difficult to compare between programmes, both at national and international levels [26–30]. To overcome this, the importance of international collaborative efforts to research interprofessional education practices has been emphasized [31] but to date, such collaborations are scarce. In particular, there is a need for theory-based research and observational methods to discover and understand the basis of interprofessional actions and interactions [7, 32]. Moreover, multiple site studies are needed to inform IPE educational design, since the heterogeneity of learning activities and practices varies with the different health care systems. Visser, et al. [33] in their systematic review, described barriers and enablers of IPE at an individual level but also at a process/curricular and cultural/organizational level of the educational programmes, while Pullon et al. [34] discussed the importance of paying attention to both individual and contextual factors for sustainable collaborative practice. This indicates a need for research approaches that allow broader perspectives considering not only the experiences of the individual, but also those of the local contexts where IPE is occurring. Recent theories on research on professional learning emphasize the importance of considering the complexity and dynamics of the practices and contexts, i.e., the social and material conditions under which the learning takes place [35–37]. A scoping review highlighted the use of socio-material approaches as a theoretical lens to understand professional learning practices in IPE and interprofessional collaboration (IPC) [38]. Using a socio-material perspective makes it possible to gain a deeper understanding of how IPE practices emerge within a clinical setting, and furthermore, to develop an understanding of complex situations such as power relationships, human resource shortages in health care, patient safety and more.

In this study, the focus is on identifying how interprofessional collaboration and learning emerge when embedded in clinical practice placements designed for such purposes. The study is designed as an international, collaborative multiple-case ethnographic study. It will involve four sites of health care clinical practice situated locally in Sweden, Norway, Australia, and New Zealand. The multiple case study ethnographic research design [39] will be used in combination with Kemmis’ theory of practice architectures (TPA) [40]. This approach will make it possible to identify similarities and differences across the four countries and different sites of IPE.

## Context of study

Each country has endorsed the WHO’s global call for Interprofessional Education and Collaborative Practice (IPECP) in different ways, which has been influenced by their national and local health care organization [2]. The local experience of teaching IPE, how the learning experience is designed, and for how long the students have an IPE clinical placement, varies between the four universities. Linköping University (Sweden) has long-standing experience of an IPE-curriculum including all health education programmes. UiT The Arctic University of Norway has a long history of IPE and builds on selected interprofessional learning activities including 13 health – and social programmes at the most. The University of Otago (New Zealand) has centrally organized IPE with a staged implementation strategy for all health and social services students to undertake IPE learning activities, while the University of Wollongong (Australia) is at an early phase of developing and implementing IPE across a variety of health and social programmes. The different contexts and establishment of IPE at the four sites make up a natural variation suitable for multiple case study research [39]. A summary of key contextual issues provides a background to each country (Table 1).

**Table 1:** Summary of key contextual issues of the four countries

	Sweden	Norway	Australia	New Zealand
Population (2021)	10.4 mill	5.4 mill	25.6 mill	5.1 mill
Income and purchasing power (top 50 counties in the world) [41]	16/50	6/50	22/50	30/50
Health expenditure as a share of GDP, 2019 [42]	10.9	10.5	9.4	9.1
Life expectancy in years (2021) [43]	83.2	83.0	83.0	82.1
Language	Swedish	Norwegian	English	English
Indigenous peoples	Sami	Sami	Aboriginal and Torres Straight Islands	Māori
Health care system	Publicly funded secondary and primary care at no or small co payment charges – the latter set by regions with some caps and exemptions. There is private care (some publicly funded care is purchased from private providers).	Publicly funded secondary health care which is mostly provided by the government. Primary care is arranged through municipal health services and has a fee for service (capped). Private health care is also available, mainly in bigger cities.	Publicly funded health care at no or reduced cost through Medicare (funded by tax). The private system includes health service providers (owned and managed privately), such as private hospitals, specialist medical and allied health, and pharmacies.	Publicly funded secondary care Partly public and user pays primary care
Introduction of IPE into pre-registration education	Dependent of each Swedish university. Linkoping University (LiU) has systematically offered IPE to Health sciences students since 1986 with a continuous development over the years. Learning objectives at a national level.	Dependent on different University/ University college in Norway. Early initiatives at UiT The Arctic university of Norway since 1990 with continuous development and implementation of a 10 ECTS for 13 health- and social programmes in 2013. IPE regulated in higher education by legislation in 2017.	Early programmes from 1975. Implementation is dependent on each University. Accreditation bodies for each health education programme are requiring IPE activities as a part of a plan to strengthen interprofessional practice in the health system.	Dependent on each New Zealand university. University of Otago has systematically offered IPE to pre-registration health sciences students since 2018 but some IPE has been offered since 2011

## Theoretical framework – theory of practice architecture (TPA)

**Fig. 1:**
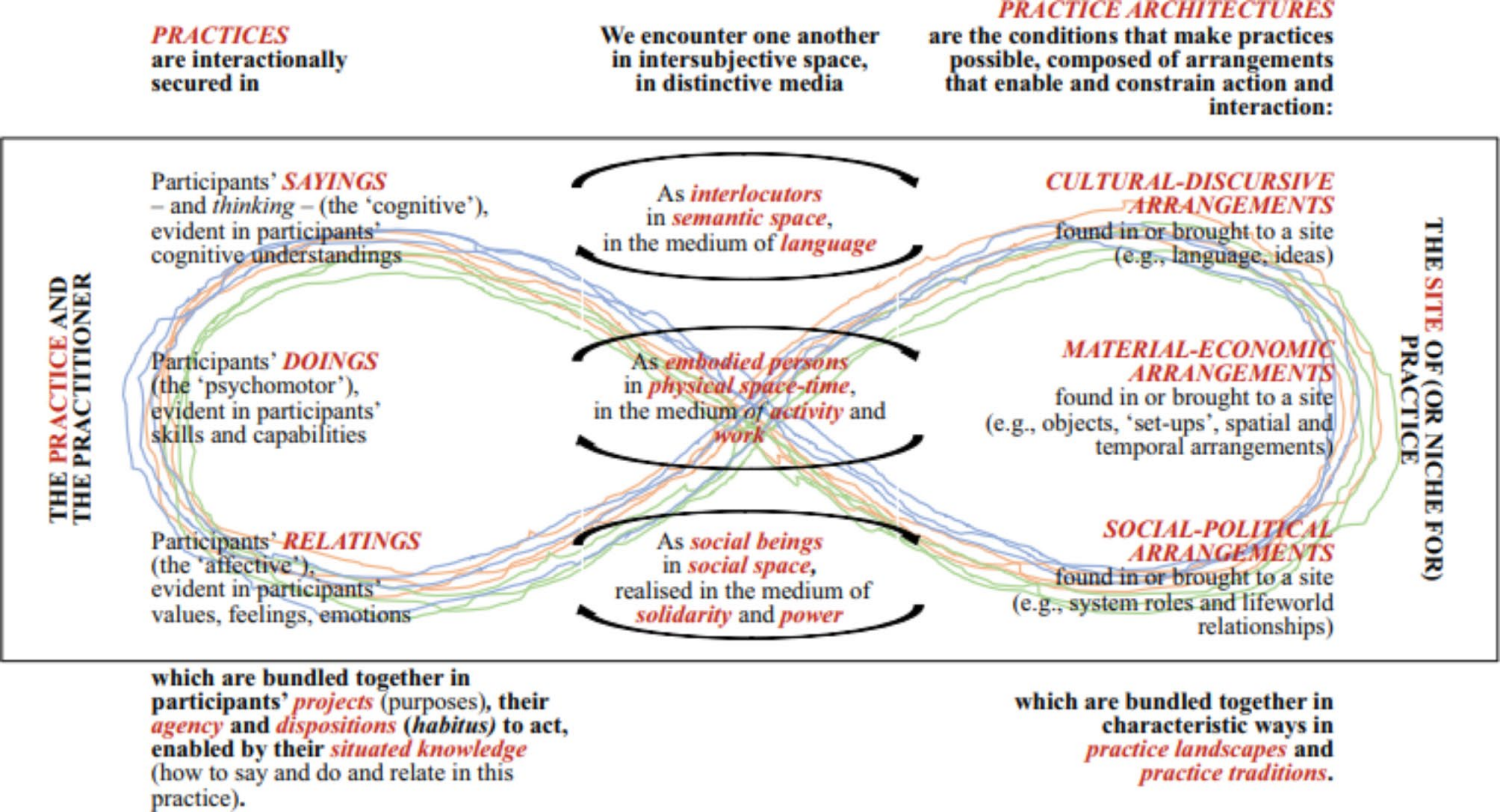
Kemmis´ theory of practice architectures (TPA) [40] p.97. (with permission from the author)

We will use a theoretical framework based on Kemmis’ TPA [36, 40] (see Fig. 1). The TPA is increasingly being used to understand professional practice and the potential to learn in new ways. [36, 37, 40]. The theoretical framework uses the three recognized practice architecture dimensions of cultural-discursive, material-economic and social-political, along with their associated elements. The cultural-discursive dimension includes the interactions, discourses, and words (‘sayings’) which make the professional practice understandable; this reveals what to say and think in or about a practice, and what it means. The material-economic dimension enables and constrains how people can act and interact in physical and material space (‘doings’); this reveals the different types of activities and work performed by the professionals within a physical environment and the way these ‘doings’ influence others in the same practice. The social-political dimension describes the relationships that form between individuals and groups (‘relatings’); this reveals how relationships between certain arrangements of professionals develop, their roles, and whether and how relations continue to exist or not [44]. The emphasis is therefore on the relationships between material arrangements and human actions and what these produce [37], and that these relationships are more, or less likely to happen, in certain circumstances [45].

According to TPA, IPE in clinical placements can be viewed as an organized set of actions and interactions embedded in a professional practice. This means that both human and non-human factors are considered. The focus of the study is the students’ sayings, doings and relatings with fellow students, patients, supervisors, and staff, in the complex dynamic and relational dimensions of practice, i.e. the social and material conditions under which the clinical placement or learning activity is arranged.

## Methods

### Purpose and aims

The aims of this research project are to identify:

1. how IPE in clinical placements emerges, evolves, and is enacted by students when embedded in local health care practices,
2. factors critical for the design of IPE for students at clinical placements across the four countries.

Four research questions (RQ) will be explored:

#### RQ1

How do interprofessional clinical placements enable students collaborative learning activities? RQ2. How do students’ sayings, doings and relatings in practice shape interprofessional collaboration and learning?

#### RQ3

What challenges do interprofessional clinical practice placements bring to established health care practices?

#### RQ4

What lessons from the case studies can inform the global discourse on interprofessional educational practice?

### Case study site selection

Each case study site has been purposively selected within each country and across the four countries (see Table 2). Purposive selection has been used to ensure maximal variation [46].

**Table 2:** Case study sites

Country	Brief summary of case site
Sweden	Two-weeks IPE in primary health care services where students from three professions (Medicine, Nursing, Occupational Therapy) undertake interprofessional activities such as planning before a patient meeting, clinical examinations, documentations and reflection sessions. Practitioners from each profession are dedicated as supervisors.
Norway	Hospital and community based primary health services where interprofessional groups of five students, drawn from a diverse range of professions including Medicine, Nursing, Physiotherapy, Psychology, Dentistry, Social Work, Social Education, Speech Therapy, Pharmacy, Dental Hygiene, Music Therapy, Occupational Therapy, and Biomedical Laboratory Science, engage with patients. These student groups participate in activities such as conversations, observations, examinations, and development of individualized care plans. Practitioners at each site are dedicated as coordinators and dialogue partners for the students. A one-day placement for each group is followed by a dialogue meeting the following week (2 h), with practitioners from the placements and a facilitator from the University.
Australia	Older persons residential care setting where students from three or more health disciplines (Medicine, nursing, social work, psychology, rehabilitation and dietetics) undertake clinical case reviews including conversations, physical examination and development of a clinical management plan. Interprofessional management plans will be developed for a number of patients over a series of days, and students will then present their findings to a multi-disciplinary professional team.
New Zealand	Five-week IPE in an isolated rural setting. Students in a group of up to eleven, including one or more from the following professions: dental, medical laboratory science, medicine, nursing, occupational therapy, pharmacy, social work, live together in shared accommodation and undertake a mix of uni-professional and interprofessional placements and learning activities. They have an onsite interprofessional facilitation team, as well as local clinical placement supervisors. Placements include primary care and rural hospital sites

## Data collection

### Methodology

Four case studies will be undertaken, one each by the local research groups based in Sweden, Norway, Australia, and New Zealand and using a common ethnographic methodology.

An ethnographic approach focuses on understanding the social processes and cultures of different contexts [47], and usually comprises a range of qualitative methods. It is recognized as a suitable research method for acquiring knowledge about how practices are arranged and interrelated within naturally occurring physical and social environments, and about the contexts in which activities and knowledge-sharing can take place [45, 48].

### Methods

The initial site visits by each respective country’s local research team will take place in late 2023 and early 2024. At each case site the researcher(s), all connected to health profession education, will use the case study observational research (CSOR) method where non-participant observation guides data collection. In the CSOR method, the direct observations of participants’ behaviours and interactions are given priority and precedence over self-reported forms of data collection, and collection of non-observational data is informed by the analysis of the observational data to enable further investigation of observations [49, 50].

Direct observation allows the researcher to see what is occurring rather than having participants describe what they do through interviews. Observations of students will follow the naturally occurring rhythm of interprofessional activities during the day. Examples of such activities are the students planning together their daily work, encounters with patients, deliberations following their work on what seems to be proper treatment and advice for the patient in question, students interactions with staff and supervisors, and their daily reflections on how they have been working together and what they have learned. Each case site is different, and the IPE learning activities is of different length and with different learning outcomes. In each case site, researchers will act as observers of interprofessional students in action and write detailed fieldnotes or record audio memos on the interactions and their context. Field notes will also incorporate the researcher’s reflections “including feelings, actions and responses to the situation” [39]. Brief informal conversations with students may be conducted during or immediately after the observations if clarity is needed about what has been observed, and these will be recorded in the field notes [47]. Non-clinical documents may be used to support the analysis, and photographs may be collected for documenting context and to aid recall. These comprehensive observations will facilitate the systematic collection of data while still acknowledging the influence and interpretations of the researcher in the data collection process. The CSOR method will make it possible to gain access to observed actions, interactions and discussions that take place between students (sayings, doings and relatings), and between students and patients, staff, and others.

In each case, the observational data and field notes (and if needed non-clinical documentation and/or photographs for context) will be immediately circulated to the local research team and reflexive feedback provided for inclusion in the analysis. Following this rapid analysis of observational data and guided by what further data is needed or needing to be corroborated, formal interviews will be booked as soon as possible with students, patients, clinical tutors, IPE teachers and others, Formal interviews (audio recorded) will be guided by a template of core questions developed by the research project team. This common template will be augmented by other questions informed by each initial case analysis. Data will be transcribed either selectively or fully; English language translation will occur where data are being analysed for comparative analysis.

### Analysis

#### Theoretical approach

Data analysis will use TPA [40] including an analysis “tool kit” [51]. The tool kit is a theory and method package to investigate practices by the systematic interpretation of the case study data. A “zooming in – zooming out” methodological approach [51], will make visible details in a specific local practice; “zooming in” allows getting close to the practices being observed (to answer RQ1 and RQ2) and then “zooming out” allows the researcher to expand their scope and look for connections between different practices (RQ3 and RQ4). The connections between practices in the research study will be identified through focusing on the three dimensions of practice architectures: the cultural-discursive, material-economic, and social-political. The agreed tool kit approach will include a layered, purposeful constant comparative analysis [52], comprising three phases of individual and collaborative activities, using English as the common language. First the systematic collection and analysis of observations and field notes of those observations and other qualitative data by each local research team, will be guided by the theoretical perspective on how students interact in relation to social and material arrangements. Second, the data in each of the four case study sites will be analysed by each local research team and verified locally and collectively; this will lead to site-specific findings. Third, comparisons will be made between the four different sites by cross-checking and developing and refining the interpretations of all the data.

#### Practical approach

Each country will follow the data collection and analysis process outlined in the methods for their case site and each case site will be analysed separately. Each local research team will have regular meetings to ensure that a reflexive, but uniform approach is undertaken as data is collected. These meetings will also include workshops for collaborative data analysis. Monthly meetings will also be held between the four countries’ project research teams as case data collection and analysis progresses and a similar reflexive process used. This will ensure the analyses of each case follows the same process and will provide assurance of mutual understanding across sites. To enable this, anonymized observational data (and fieldnotes), interview data and photographic or document extracts will be shared, analysed and reviewed in workshops. Following completion of each case study in the four different countries, a cross-case process [39] will be undertaken. Each local research teams will first have undertaken the primary analysis, combining data from fieldnotes and interview transcript generating preliminary themes to identify the sayings, doings and relatings are emerging and connected in the efforts of collaborate around the patient. As the findings are first collated, observed aspects from students’ sayings, doings and relatings, projects and dispositions will be revealed. As a second layer of analysis, the findings will analytically be connected to practice architectures, such as the cultural-discursive, the material-economic and the socio-political arrangements. The use of a common scheme for how to document the analysis is important for comparative reasons and indicate points for shared analyses across the research teams to consider the respective results, identify similarities and differences across the four sites, and explore any learning principles that might apply to IPE internationally. It is intended for each country to use the same processes to anonymize, catalogue and code the transcribed data. The research agreement also includes a process to enable sharing of selected portions of data and coding software databases using password-protected systems [53].

### Ethical approval and consent

The research group in each country will be responsible for (1) seeking ethical approval for their respective case, (2) gaining consent from each local site to undertake the respective case study, (3) establishing rules for storage of the data. The following countries have received ethical approval to proceed: Sweden (Dnr 2023-02277-01), Norway (No.889163), New Zealand (No. H23/035), Australia (underway).

### Establishing trustworthiness

The following processes and definitions proposed by Korstjens and Moser [54] based on Lincoln and Guba [55] will be used to ensure trustworthiness in the implementation of this study (Table 3).

**Table 3:** Summary of measures to ensure trustworthiness

Quality criteria	Strategy
Credibility: Confidence that can be placed in the trustworthiness of the research findings	***Prolonged engagement***: the researchers will undertake between 10–20 h of non-participant data collection in each site, observing students in different types of learning activities. They will ensure they are familiar with the site, are accepted by students and others and understand the local context of health care delivery. ***Persistent observation***: Observations are comprehensive and focused in order to capture all elements of the interactions. ***Triangulation***: The observational data will be referenced and guide further non-observational qualitative data collection to expand, corroborate and thus triangulate in each case study.
Transferability: The degree to which findings can be transferred to other contexts	***Thick description***: Providing data examples of behaviour, experience and context including sayings, doings and relatings. Each country will take turns to present their feasibility study data and in due course the case data to ensure there is common agreement in how the tool kit approach is being implemented. When data is presented, Sweden and Norway examples will be translated into English language.
Dependability (Stability of findings over time) Confirmability (degree to which findings could be confirmed by other researchers)	***Preparation***: the research team have met monthly since 2021 when this project was first proposed. They have undertaken training in 2023 in the observational method and using the theory of practice architectures through a series of Zoom workshops led by an external expect advisor where video recordings were watched, observations recorded and then discussed. ***Audit trail***: Regular meetings will continue in each country and across the four countries. A record will be kept of every decision made throughout the research and will be recorded as meeting minutes. This record will be kept for each country’s case and for the four countries as a whole.
Reflexivity: Continuous, collaborative and multifaceted practices whereby researchers overtly critique, appraise and evaluate if and how their subjectivity and context influences the research process.	***Process***: prior to and throughout each case, researchers will explicitly examine their pre-conceptions and biases (e.g., conceptual lens, assumptions, values) and consider how they may influence data collection or analyses and other research decisions [56, 57]. The same process will occur for the four-country research team as a whole; the latter will also include influences which may occur because of each country’s views on and resourcing of, health and health care delivery, or other contextual or environmental influences (pandemic, war). ***Record***: *The process of undertaking reflexivity will be recorded at each research meeting and will be used as data.*

#### Timeline

A timeline for the research project has been established (Table 4).

**Table 4:** Timeline of research

2023–2024	Application for ethical approval in each county Methodology and analysis training for the research team Consent obtained to undertake the case study in each country. Feasibility study of the methods in each country and adaptations if necessary.
2024–2025	Data collection for each case, continuous local analysis as well as cross-country analysis. Participating at conferences.
2025–2026	Continued analysis and publications. Participating at conferences.
2026–2027	Disseminations and Publications. Participating at conferences.

## Discussion

This research project is innovative as it takes an international approach to a globally identified educational challenge regarding methods to design and implement IPE in clinical practice settings. The approach, using case studies in four different countries, will explicitly acknowledge that educational phenomena and learning are contextually bound and situated and that although each country involved is different, common learning can be gained.

It is hoped that the four case studies will lead to new understanding and conceptualization of how IPE can be arranged within and across diverse contexts, languages, and local conditions. Furthermore, the cases may establish some of the challenges interprofessional clinical placements for students may bring to existing or established health care practices.

It is recognized however that while each country’s case will lead to new understanding for that country, it may be challenging to establish cross country learning as the context of each may be very different. Although English language will be adopted for communication, there may be subtle differences in how language is used and understood between English and non-English speaking countries, as well as between English speaking countries.

Taking account of local context as well as developing joint findings will be a challenge. The TPA will give opportunities to identify and analyse how students´ interprofessional clinical activities are embedded in the complex practice of routine health care at a local level within each country, and between countries. The theory will make it possible to capture how the students act in practice and how they relate to each other in clinical placements. It is hoped it will also show how clinical and interprofessional practices are influenced through the three different dimensions (cultural-discursive, material-economic, and social-political) and if these may construct, enable, or constrain practice work and knowledge-sharing. Possible examples may include: (1) the influence of a discipline’s language or discourse; the way of speaking that forms the framework for understanding themselves and others, (2) the arrangement of a health care setting; the way the environment influences where students can meet and work together (e.g. patient care rooms, rooms used for ward rounds and corridors), and (3) the development of relationships; the way social norms and political influences impact on relationships between different disciplines and groups [40, 45]. It is possible when the analysis progresses that the three dimensions referred to above may show nuanced differences between countries which previously have been difficult to articulate and account for.

Undertaking this international collaborative research is important for IPE research going forward. International collaborative research projects in IPE are rare but have been recommended for the consolidation and growth of the IPE research knowledge base [31].

The design of IPE in clinical placements should be informed by evidence and best practice. This includes using theoretical approaches which can be replicated or further developed, such as the TPA.

This research will advance a model of IPE based on TPA. It will provide new understanding and conceptualization of how IPE can be arranged across diverse contexts and local conditions, but with a common aim to provide collaborative practice-ready graduates able to respond to the increasing healthcare demands of the future.

Therefore, the broader impact of the proposed study is expected to contribute to: (1) the local and international educational IPE community regarding design of IPE in clinical practice, and (2) the international IPE research community regarding how IPE in practice can be collaboratively researched.

## Data Availability

Selected data will be reported in the Results section but will not be available as datasets.

## Abbreviations

CSOR: Case Study Observational Research
IPE: Interprofessional Education
IPECP: Interprofessional Education and Collaborative Practice
IPC: Interprofessional Collaboration
TPA: Theory of Practice Architectures
WHO: World Health Organisation
